# U. S. Pharmacopœia

**Published:** 1863-09

**Authors:** 


U, S, Pharmacopœia.—We had hoped to have been able to present a notice of this work, which would have been published ere this, but for an expected delay of two weeks, arising out of the necessity of getting type made for expressing the accentuation in the index, as directed by the Convention at Washington. The Chairman of the Committee having objected to the appearance of any notice prior to publication, we are compelled to postpone any commentary on the Pharmacopœia until our September issue. Meanwhile we have every reason to believe that the book will be published about the middle of July.—*American Journal of Pharmacy.*

